# Effective Stimulation of the Biotechnological Potential of the Medicinal White Rot Fungus: *Phellinus pini* by Menadione-Mediated Oxidative Stress

**DOI:** 10.1007/s12010-014-1064-2

**Published:** 2014-08-03

**Authors:** Magdalena Jaszek, Katarzyna Kos, Anna Matuszewska, Marcin Grąz, Dawid Stefaniuk, Monika Osińska-Jaroszuk, Monika Prendecka, Ewa Jóźwik, Krzysztof Grzywnowicz

**Affiliations:** 1 Department of Biochemistry, Maria Curie-Skłodowska University, 19 Akademicka Street, 20-033 Lublin, Poland; 2 Chair and Department of Human Physiology, Medical University, 11 Radziwiłłowska Street, 20-080 Lublin, Poland

**Keywords:** *Phellinus pini*, Menadione, Oxidative stimulation, Stress response, Manganese peroxidase, Chitinase

## Abstract

The effect of menadione (MQ; 2-methyl-1,4-naphtoquinone), a superoxide-generating agent, on the natural biodegradation system in the medicinal white rot fungus *Phellinus pini* was determined. While measuring the activities of extracellular manganese-dependent peroxidase (MnP) and intracellular chitinase, it was found that the application of MQ (0.75 mM) distinctly stimulated the activities of these enzymes in comparison to the control values (without MQ). Using the capillary electrophoresis (CE) method, an increase in the extracellular oxalic acid (OXA) concentration was detected during the first days after the addition of MQ. It was observed that the rate of intracellular proteolysis at pH 3.5 evidently decreased under oxidative stress conditions. Contrary to these results, the activities of serine proteases at pH 9.5 measured against fluorogenic peptide substrates distinctly increased in stressed cultures. The MQ treatment also caused an evident increase in the catalase (CAT) activity, as well as the levels of superoxide anion radicals (SORs), formaldehyde (FA), and phenolic compounds (PHC) in the experimental cultures. The results obtained confirm that prooxidants may find application as an effective way to stimulate biotechnological production of MnP and chitinase by white rot fungi.

## Introduction

Recently, the white rot *Basidiomycota* have received increasing attention from researchers as an applicable source of natural components including low molecular compounds, proteins, polysaccharides, or polysaccharide–protein complexes for industry, food processing, or manufacture of drugs. The production of natural bioactive constituents is a growing field of the contemporary biotechnology [1, 2]. *Phellinus pini* from the family *Hymenochaetacae* is a widespread red color mushroom that frutifies over the stem of *Pinaceae*, *Cupressaceae*, etc. [3]. It can be an interesting source of potentially medicinal substances such as enzymes, polysaccharides, or ceramides [4]. The extract obtained from the *P. pini* fruiting body has been shown to possess antitumor, immunostimulating, antiviral, antibacterial, and hypocholesterolemic activities. Additionally, two resinous natural compounds that inhibit NO production were detected in the *P. pini* fruit body [5–8].

Development and reproduction of organisms in the natural environment can be regulated by various types of stressful conditions [9]. The stress-tolerant (S-selected) strategies evolved by fungi allow them to colonize the niches of some extreme environmental conditions. [10]. This type of life way is observed in a majority of wood-decaying white rot *Basidiomycota*. Normally, the characteristic course of their life cycle is strictly connected with a relatively high concentration of their self-produced reactive oxygen species (ROS), which might be a key factor, e.g., initiating wood degradation processes [11, 12]. The level of ROS generated during the cellular metabolism can be elevated by external stress factors, such as an increase or decrease in culturing temperature, treatment with redox-cycling compounds, or presence of metal ions. When the internal balance between the enzymatic and nonenzymatic antioxidants is disrupted, a phenomenon of oxidative stress can be observed.

In response to surrounding environmental factors including excess of ROS, these fungi secrete a broad spectrum of the above-mentioned ligninolytic, hydrolytic, or antioxidative enzymes and low molecular derivatives like phenolic compounds, oxalic acid, formaldehyde, or ROS [12–14]. Apart from their synergistic action in the degradation of biopolymers, the specific ligninolytic enzymes, i.e., peroxidases [manganese-dependent peroxidase, MnP (EC 1.11.1.13); lignin peroxidase, LiP (EC 1.11.1.14); or laccase, LAC (EC 1.10.3.2)], are often described in the literature as being very interesting for modern industry and biotechnology (e.g., biotransformation of environmental pollutants, decolorization of textile dye wastes, pulping and bleaching processes, food processing, medicine) [15–18]. According to the available data, LAC and MnP may also act as an important element of the general fungal response to changes in temperature, presence of cadmium, or proxidant treatment [12, 13, 19–22].

Hydrolytic enzymes are an important part of the fungal metabolism. These include chitinases described as defense against chitin-containing pathogens important for nutrition, morphogenesis, and interspecific mycelial interactions between different fungal strains. These enzymes are produced by a number of organisms, including bacteria, fungi, plants, and yeasts. Chitinases (EC 3.2.1.14) catalyze the hydrolysis of chitin into smaller chitooligosaccharides. Some of them, for example (GlcNAc) 6 and (GlcNAc) 7, have been reported to possess possible antitumor activity. Due to the variety of physiological functions, chitinases have found several applications [12, 23].

Contrary to the biodegradation processes conducted by the white rot fungi, only few reports described their oxidative stress response system including the possible role of ligninolytic and hydrolytic enzymes or low molecular weight compounds in this type of defense [24]. This is the reason why the main question of the present study was whether the stress stimulation, like other inducers, could be an effective way to increase the biosynthesis of fungal biotechnologically applicable and bioactive compounds and to stimulate their natural potential of biodegradation. Because of its specific quinone structure, a superoxide-generating agent—menadione (MQ)—was considered as a very interesting prooxidant, especially for white rot fungi [25]. Intermediate metabolites produced during lignin biodegradation may include phenolic compounds, which are enzymatically transformed to quinine forms. The quinone-redox cycle catalyzed by LAC is one of the most important sources of ROS, e.g., the superoxide anion radical (SOR), hydrogen peroxide, and hydroxyl radical (byproduct of Fenton-type reactions) [26]. The prooxidants or heat shock conditions can also control the biosynthesis of MnP, an enzyme that catalyzes the Mn-mediated oxidation of phenolic lignin compounds [12, 13, 27, 28].

Based on the above information, our first aim was to determine the extracellular activities of LAC and MnP in the *P. pini* cultures treated with MQ. The next step of the experimental cycle was to determine whether the addition of MQ really caused overproduction of SORs and the other low molecular stress marker formaldehyde (FA) under these experimental conditions. The level of phenolic compounds (PHC) and oxalic acid (OXA) as targets employed in ligninolytic enzyme reactions and fungal stress response mechanisms was also measured. Furthermore, in the experiments described above, the activities of SOD and CAT, the well-known enzymatic antioxidants, were measured in the stressed and control cultures. The effect of MQ addition on the activities of proteases and chitinase was also determined in the present work.

## Materials and Methods

### Strain, Media, Growth, and Oxidative Stress Conditions

The *P. pini* (Brot.; Fr) Ames strain was obtained from the culture collection of the Department of Biochemistry, M. Curie-Skłodowska University in Lublin, Poland [29]. The fungal cultures were stored on 2 % (*w*/*v*) malt agar slants. For inoculation, fungal agar plugs (approximately 0.5 cm^2^) were cut and put into the mineral medium prepared according to Fahreus and Reinhammar (FR) [30]. The fungal cultures were performed as described previously [13]. The mycelium was collected and homogenized in a Warning blender. Experimental stationary cultures were inoculated with 2.5 % (*v*/*v*) of homogenized fungal material and incubated in 25-ml Erlenmeyer glass flasks with 10 ml of the FR media at 25 °C for 14 days. Since the lignin-modifying enzymes and many biotechnologically important compounds are expressed mainly during the secondary metabolic phase of fungal growth [31], 14-day-old stationary cultivated, idiophasic cultures of *P. pini* were treated with different concentrations of menadione solution (0.5–2.5 mM). The beginning of the idiophase was determined according to Jennings and Lysek [32]. The proper experimental concentration of MQ was established, based on the MnP, LAC, and chitinase activities. The most significant changes were observed in the presence of 0.75 mM of MQ (final concentration in the medium). In the case of stationary cultivated fungal cultures, the portions of MQ must be added immediately to the culture fluid.

### Preparation of Fungal Material

Fungal cultures were harvested periodically during 10 days (at 1, 5, and 10 day) after chemical treatment. Mycelia were separated from the culture fluid using the Miracloth (Calbiochem), washed out twice with distilled water, and homogenized in phosphate buffer (pH 7.4) by a glass Potter’s homogenizer at 4 °C. After centrifugation (15 min, 10,000×*g*), portions of the crude supernatant were frozen. The intracellular extracts and extracellular fluids of the menadione-treated and control cultures were and used for the experiments [13].

### Enzyme Activity Assays

LAC activity was determined based on the oxidation of syringaldazine (4-hydroxy, 3,5-dimetoxybenzaldehyde) and expressed in nanokatals per milligram of protein [33]. Extracellular MnP activity was determined according to the Wariishi et al. method based on the rate of Mn^3+^-malonate complex formation at 270 nm [34]. Specific MnP activity was expressed in nanokatals per milligram of protein. The activity of chitinase was determined using 4-methylumbelliferyl-b-N,N0,N00 triacetychito-trioside [MUF-3] as the substrate [35]. The reaction mixture contained 0.05 ml of the enzyme sample, 0.1 ml of 100 mM phosphate buffer (pH 6.5), and 50 μl of substrate solution. The reaction was started by addition of the substrate solution at 20 °C. After incubation at 20 °C for 15 min, the reaction was stopped by the addition of 0.2 M Na_2_CO_3_. The amount of released 4-methylumbelliferyl (4-MU) was measured using FluoroMax-2 equipment (Horiba, Japan) (excitation, 390 nm; emission, 460 nm), and the specific activity was expressed as the amount of 4-MU released per min per mg of protein. Catalase activity was determined according to the Aebi method [36] based on the amount of hydrogen peroxide degraded by the enzyme during the incubation time (30 s). The rate of absorbance changes were recorded at 240 nm, and the specific activity of CAT was expressed in nanokatals per milligram of protein. Serine proteinase activities were measured using the fluorogenic substrates: Z-Gly-Gly-Leu-AMC for subtilisin-like activity and Bz-Arg-AMC-trypsin-like activity [37]. The rate of conducted enzymatic hydrolysis was measured by the release of aminomethyl coumarin (AMC) from the substrate as emission at 440 nm upon excitation at 340 nm. The specific activities of serine proteinases were expressed as a micromolars of released AMC per milligram of protein.

### Assay of the Relative Level of Superoxide Anion Radicals

The SOR level measurement was done according to the method for rapid detection of superoxide anion generation in fungal material [38]. The relative level of SOR was estimated spectrophotometrically based on the detection of superoxide-induced formation of formazan from nitrotetrazolium blue (NBT) under alkaline conditions, as described previously [13]. The reaction mixture containing 3 ml of distilled water, 0.05 ml of 1 M NaOH, 0.1 ml of 5 mM NBT solution, and 0.1 ml of the sample was incubated (30 min at 20 °C); absorbance was measured at 560 nm. The alkaline conditions are to prevent the precipitation of dark-blue formazan for about 40 min.

### Formaldehyde Assay

The formaldehyde level was measured spectrofluorimetrically with Nash reagent (exCitation, 410 nm; emission, 510 nm) using FluoroMax-2 equipment [39]. The emission data for diacetylodihydrolutidine formation were compared with the calibration curve and expressed as micromolars per microgram of protein.

### Detection of Oxalic Acid Using CE

The CE analyses were performed on Thermo Capillary Electrophoresis, Crystal 100 (Thermo Separation Products, San Jose, USA). Before the assays, the samples of culture fluids were prepared by ultrafiltration on a Microcon Centrifugal Filter (3000 NMWL) designed by Millipore. The separations were conducted as described previously [13] in a fused silica capillary with a total length of 75 cm (50 cm to detection window) and an inner diameter of 500 nm (voltage applied was −25 kV, capillary temperature was maintained at 25 °C). After hydrodynamic injection (for 0.5 s), the detection was provided at 210 nm. The buffer solution (pH 7.0) contained phtalic acid (5 mM), cetyltrimethylammonium bromide (CTAB, 0.26 mM), and methanol (0.5 %) in MilliQ water [40]. The samples, as well as the reagents used, were filtered through 0.22-μm syringe filters before the assays. Peak identification was performed by comparison with the standard solution, and the concentration of oxalic acid was calculated and expressed in millimolars.

### The Concentration of Phenolic Compounds

The extra- and intracellular concentration of phenolic compounds (-hydroxyl, -metoxy phenolic acids) was determined with diazosulfanilamide (SA) according to DASA test [41]. The reaction mixture contained the following components: 0.1 ml of SA (1 % SA in 10 % HCl), 0.1 ml of 5 % NaNO_2_ solution, and 0.1 ml of the sample. After stirring, the samples were neutralized by the addition of 1 ml of 20 % Na_2_CO_3._ The changes in absorbance were recorded at 500 nm and compared with the proper calibration curve (*y* = 6.85*x*−0.0218, *R* ^2^ = 0.999).

### Protein Assay

The protein concentration was determined with the Coomassie brilliant blue (G-250) dye-binding method [42] using a Bio-Rad dye solution with bovine serum albumin as the standard. The parameters presented in the paper refer to the amount of protein in the proper sample.

### Analysis of SOD and Proteolytic Activities by Native PAGE Electrophoresis

The supernatant of homogenized mycelia and the extracellular culture fluid were concentrated and separated by ultrafiltration using Microcon Centrifugal Filter Units, 3000 NMWL designed by Millipore. Subsequently, 15 μg of extra- or intracellular proteins were deposited per lane (native polyacrylamide gels). The separation of proteins was conducted 4 °C at 145 V. In the case of SOD, the 12.5 % native polyacrylamide gels were used and the bands of enzyme activities were visualized according to the method of Beyer and Fridovich [43]. The changes of proteolytic activities were determined using 10 % native polyacrylamide gels with 0.3 % of casein as a reaction substrate [44]. After electrophoretical separation, the gels were incubated for 18 h at 37 °C in 0.1 M citrate-phosphate buffer (pH 3.5). Coomassie brilliant blue (R-250) staining was used for detection of bands of proteolytic activities.

### Statistical Analysis

Each result is expressed as mean ± SD from three experiments (*n* = 3). The results were subjected to analysis by ANOVA procedure, and means were compared using post hoc Tukey’s test. All calculation were conducted using the Excel program (Microsoft Office 2010 package). Only values with a significance of *p* ≤ 0.05 were reported as different.

### Chemicals

Most reagents and substrates used in the experiments were obtained from Sigma (St. Louis, USA) and POCH (Poland).

## Results and Discussion

Fungi belonging to white rot basidiomycetes possess a unique ability to completely depolymerize and mineralize both wood polysaccharides and lignin in a simultaneous or selective way [45]. Since microbial enzyme production is a growing field of biotechnology, fungi are also very promising from the practical point of view [14]. Their specific enzyme system consists of laccase (phenoloxidase), manganese peroxidase (MnP), and lignin peroxidase (LiP), which they secrete [12]. Although the primary role of these enzymes is biodegradation of lignin, the broad spectrum of their activities and highly oxidative and nonspecific nature gives them a variety of other biological functions.

Since it was found that the addition of 0.75 mM MQ solution caused the most significant changes in both the extracellular activities of MnP and intracellular activities of chitinase, this concentration of the prooxidant was used for the all subsequent experiments. LAC activity measurements showed that its level was slightly higher in the cultures after MQ addition, but, in general, it was very low (data not shown). Previously, some authors have reported no LAC activity in some fungal cultures, e.g., *Phellinus flavomarginatus* growing on *Eucalyptus grandis*. In the case of *P. pini*, MnP seems to be probably the main ligninolytic activity [9]. The unique mechanisms of stress response of white rot fungi confirms the fact that similar doses of MQ (0.5 mM) added to the culture of organisms from other groups, e.g., *Penicillium chrysogenum* evidently inhibited its metabolic processes [46].

**Fig. 1:**
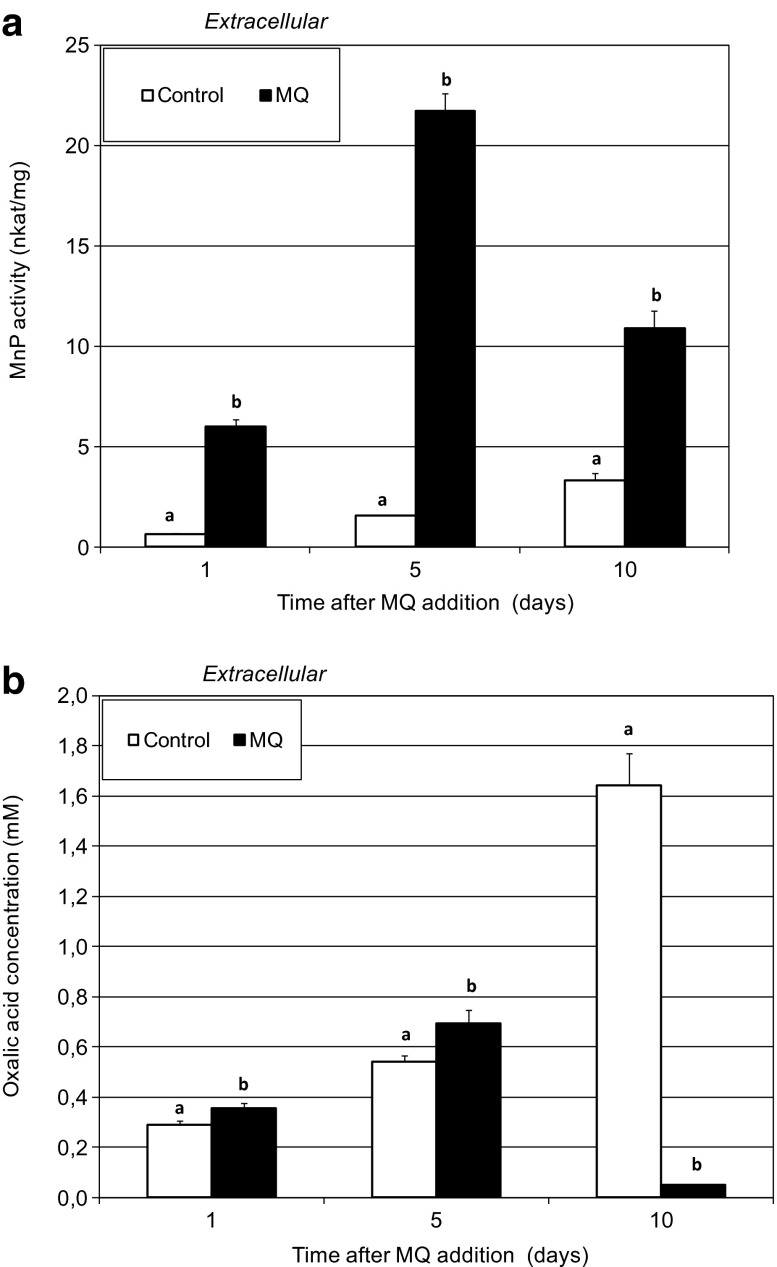
Changes in extracellular MnP activities (expressed in nkat mg^−1^ protein) (**a**) and oxalic acid concentrations in the culture medium fluid (expressed in mM) (**b**) after addition of 0.75 mM of MQ solution to the 14-day-old fungal cultures of *P. pini*. Culture medium samples were collected for 1, 5, and 10 days after MQ addition. Data are mean ± SD for three measurements (*n* = 3). Values with *different letters* are significantly different (*p* ≤ 0.05)

The results presented in this paper show a very significant increase in extracellular MnP on days 1, 5, and 10 after MQ application (Fig. 1a). The highest values were recorded 5 days after application of the prooxidant (10 times higher in comparison to the control samples). MnP, which catalyzes oxidation of Mn^2+^ to Mn^3+^ in the presence of H_2_O_2_, is proposed as the key enzyme in the transformation of lignin or aromatic xenobiotics. Available data show that the production of MnP may be enhanced in response to stress including interspecific mycelial interactions associated with overproduction of ROS causing oxidative stress [12, 47]. The evidence for the stimulation effect of 1 mM of MQ on MnP activity was found previously in stationary cultivated *Fomes fomentarius* and *Tyromyces pubescens* strains [13]. It was detected that MnP activity increased in response to abiotic stress factors e.g. elevated temperature [19], chemically-induced oxidative stress [13], or presence of cadmium [21], which resulted in oxidative stress and ROS accumulation.

Since the regular action of MnP requires stabilization of Mn^3+^, e.g. by some organic chelators, the extracellular concentration of oxalic acid was measured and compared with enzyme activity [48]. CE analysis of the oxalic acid level showed an increase in the extracellular oxalic acid concentration correlated with higher MnP activity on day 1 and 5 after MQ addition. The level of OXA dramatically decreased 10 days after the stress (Fig. 1b). Several reasons can be mentioned for this type of reaction. An important fact is that OXA can take part in nonenzymatic initiation of lignocellulose biodegradation through ROS formation, buffering fungal vicinity, cooperation with oxidative enzymes, and stabilization of formation of Mn^3+^ ions by chelation. Additionally, it can be oxidized by MnP [49]. Besides chelating of unstable Mn^3+^ ions, it also provides a source of H_2_O_2_ for peroxidase action [50]. The low level of OXA after the MnP activity peak may probably be a consequence of enzymatic catalysis. Previously, it was found that the level of OXA dramatically decreased also in *T. pubescens* and *F. fomentarius* cultures 5 day after MQ addition. A reverse correlation between OXA concentration and activity of MnP was observed in that case [13]. In plants, one of the defense mechanism in response to pathogens is the oxidation of oxalate by oxalate oxidase (EC 1.2.3.4). Enhanced secretion of OXA in the presence of metals observed in fungi suggests its possible role also in metal detoxification mechanisms [49–52]. The correlation between the increased MnP activity and the increased level of oxalic acid at 1 and 5 days after MQ addition may indicate cooperation between the factors described in response to oxidative stress.

The quinone structure of menadione is the basis for its participation in the redox cycling reactions in biological systems. After one-electron transfer, it forms semiquinone radicals that can immediately reduce O_2_ [25]. Two mechanisms are known as the cause of quinone toxicity: formation of complexes with biological molecules and cyclic overproduction of superoxide and other ROS produced by redox cycling. In white rot fungi, quinones may be oxidized to a semiquinone form, e.g., by LAC. This reaction is the base for the production of SORs and possible formation of H_2_O_2_ as a result of SOR dismutation [13, 53]. Exposure of *P. pini* cultures to MQ increased distinctly the level of SOR in the homogenate of mycelia as well as in the culture fluid (Table 1). The highest level of extracellular SOR was noted 1 day after MQ addition, whereas in the case of intracellular values—on days 1 and 10 after the prooxidant treatment.

**Table 1:** Changes in the specific intracellular CAT activities and in the level of SOR, FA, and PHC in *P. pini* mycelia and culture fluid caused by MQ-mediated oxidative stress

Time after MQ treatment (days)						
	1		5		10	
	Control	MQ	Control	MQ	Control	MQ
Intracellular						
CAT^a^*	112 ± 3.2a	897 ± 4.9b	185 ± 5.2a	1,748 ± 11b	112 ± 8.0a	768 ± 9.5b
SOR^b^*	201 ± 5.2a	584 ± 6.8b	393 ± 4.3a	390 ± 3,2a	193 ± 7.4a	425 ± 5.6b
FA^c^*	2.55 ± 0.14a	15.3 ± 0.44b	2.60 ± 0.07a	16.7 ± 0.30b	2.58 ± 0.19a	31.4 ± 0.82b
PHC^d^*	171 ± 2.3a	1,180 ± 8.5b	193 ± 3.5a	806 ± 5.6b	429 ± 4.8a	2,189 ± 10.5b
Extracellular						
SOR^b^*	17 ± 0.5a	586 ± 8.5b	84 ± 2.3a	241 ± 4.8b	48 ± 1.5a	158 ± 2.8b
FA^c^*	1,380 ± 11.1a	1,718 ± 18b	990 ± 12.3a	914 ± 45.4a	1,099 ± 26.7a	1,210 ± 16.8b
PHC^d^*	122 ± 3.8a	391 ± 5.6b	65 ± 2.0a	247 ± 5.8b	136 ± 2.9a	172 ± 4.2b

The given values (±standard deviation) are averages of three independent experiments performed in triplicate. The values within the lines followed by different letters (for particular day after oxidative stimulation) are significantly different (*p* ≤ 0.05).

^a^Catalase activities were expressed in nkat mg^−1^ of protein

^b^Relative levels of superoxide anion radicals (SOR)

^c^Concentration of formaldehyde (FA) (μM/μg)

^d^Phenolic compounds are given in % of the of the beginning control values of samples collected just before prooxidants addition

Effective interplay between enzymatic and nonenzymatic antioxidants is responsible for the cellular antioxidative protection system. The rebuilding of the intracellular metabolism observed under the MQ-mediated stress conditions caused the overproduction of FA proposed as a stress marker. The highest values of FA were observed 10 days after the addition of the chemical (Table 1). A similar reaction was noted in *T. pubescens* and *F. fomentarius* under oxidative stress conditions [13]. The most distinct alterations in the extracellular level of FA were observed on days 1 and 10 and in the intracellular level on 10 day after the stress (Table 1). A summary of the results obtained seems to confirm the earlier thesis that FA can be a marker that is produced by cells (also in the case of white rot fungi) as a reaction to the presence of several stress factors [13, 54, 55].

**Fig. 2:**
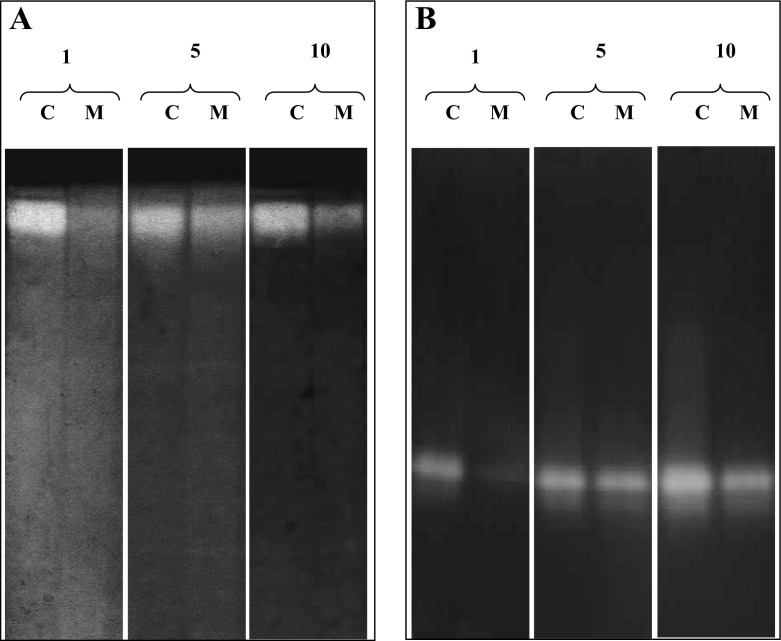
Native PAGE electrophoresis of SOD activities in the mycelia (10 % polyacrylamide gels, stained according to Beyer and Fridrovich) (**a**) as well as acid intracellular proteolytic activities (**b**) of *P. pini* MQ-treated cultures (10 % polyacrylamide gels with 0.3 % of casein, 3.5). Samples were collected at 1, 5, and 10 days after MQ addition (*C* control samples, *M* MQ-treated samples)

Superoxide dismutase, which eliminates SOR in different cellular compartments, is an important antioxidant widespread in the world of living organisms [56]. Contrary to the earlier data [13], the addition of MQ to the *P. pini* cultures decreased the electrophoretically detected activity of SOD 1, 5, and 10 days after the stress (Fig. 2a).

An important SOR dismutation product—hydrogen peroxide—is needed by fungal peroxidases for the biodegradation in wood-decay processes. For removal of an excess of this toxic molecule, cells have evolved a specific enzyme—catalase [56]. Our experiment showed that MQ evidently enhanced CAT activity in *P. pini* mycelia throughout the experiment (Table 1). The highest values were detected on day 5 after the prooxidant treatment (9.4 times higher in comparison to the control samples). A similar effect was observed in MQ-treated *F. fomentarius* mycelia [13]. The antioxidative capacity of fungal cells was elevated by the production of stress-induced phenolic compounds. An increased level of PHC was observed in the homogenate of mycelia as well as in the culture fluid (Table 1). A similar correlation was observed in *Trametes versicolor* under oxidative stress caused by addition of paraquat [22].

The final step of the presented experiments was to determine the effect of oxidative stimulation on activities of selected hydrolytic enzymes in the mycelia and in the culture fluid. Cellulases, pectinases, xylanases, chitinases, or proteases are hydrolytic white rot enzymes whose activities are most frequently detected [14, 57].

**Fig. 3:**
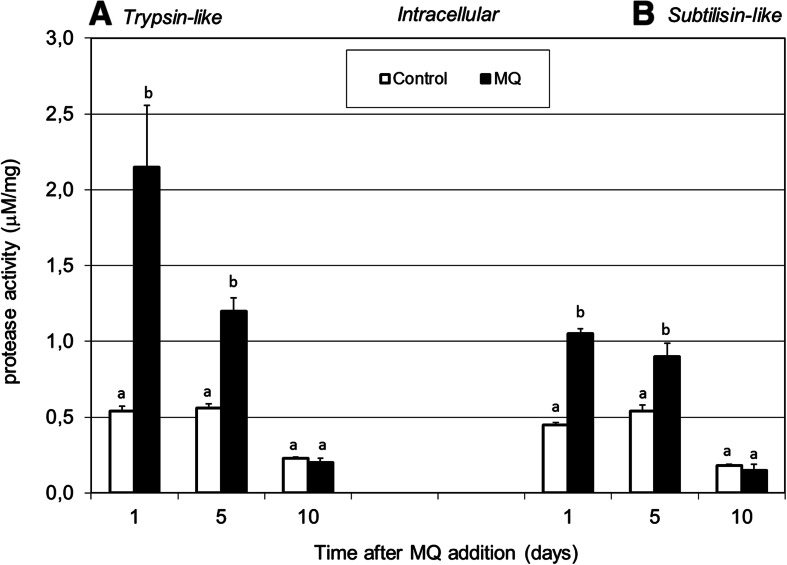
Changes in intracellular trypsin-like protease activities (expressed in μM mg^−1^ protein) (**a**) and subtilisin-like protease activities (**b**) in the fungal mycelia after addition of 0.75 mM of the MQ solution to the 14-day-old fungal cultures of *P. pini*. Fungal mycelia were collected at 1, 5, and 10 days after MQ addition. Data are mean ± SD for three measurements (*n* = 3). Values with different letters are significantly different (*p* ≤ 0.05)

**Fig. 4:**
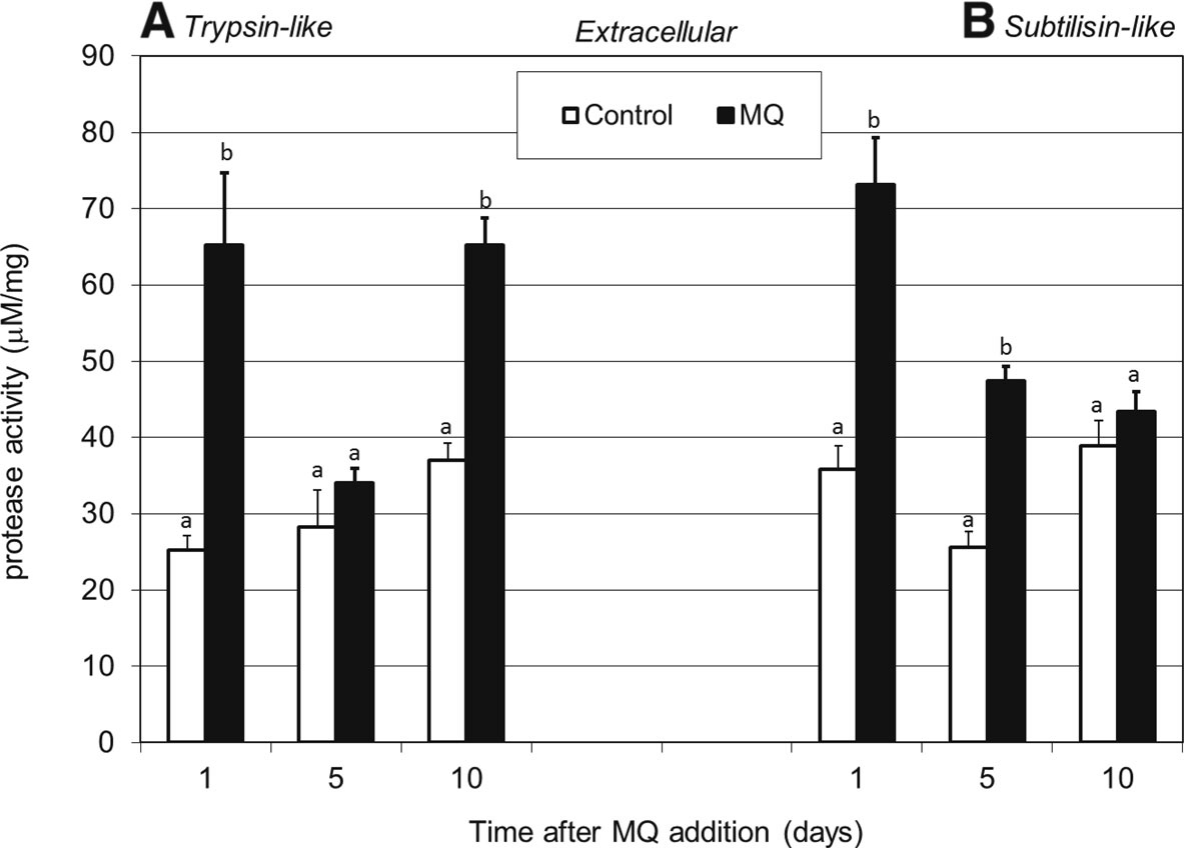
Alteration of extracellular trypsin-like protease activities (expressed in μM mg^−1^ protein) (**a**) and subtilisin-like protease activities (**b**) in the culture medium fluid after addition of 0.75 mM of MQ solution to the 14-day-old fungal cultures of *P. pini*. The culture medium samples were collected at 1, 5, and 10 days after MQ addition. Data are mean ± SD for three measurements (*n* = 3). Values with different letters are significantly different (*p* ≤ 0.05)

It is well known that many biological functions, e.g., the signaling cascade, lysosomal degradation, or digestion of nutrient substance, are based on proteases. Continuous protein turnover is involved in most biological processes in living organisms, especially as adjustment to various types of stress factors, e.g., removal of damaged proteins or activation of stress proteins [13]. Determination of the intra- (Fig. 3) and extracellular (Fig. 4) rate of proteolysis under MQ-induced oxidative stress conditions at pH 9.0 showed a significant increase in the activities of trypsin- and subtilisin-like serine proteases. The zymographic analysis of proteolysis at pH 3.5 demonstrated a decrease in the intracellular protease activities in MQ stressed cultures in comparison to the control (Fig. 2b). A reverse correlation was observed between the proteolysis at pH 3.5 and at pH 9.0 in *P. pini* under oxidative stress. Available literature data suggest a very important role of intracellular proteolysis in the response of white rot fungi to cadmium treatment, nitrogen starvation, or presence of prooxidants [13, 24, 58]. The results obtained seem to confirm that the control of continuous protein turnover is strictly involved in basic cellular functions, especially as an adjustment to stress response [24].

**Fig. 5:**
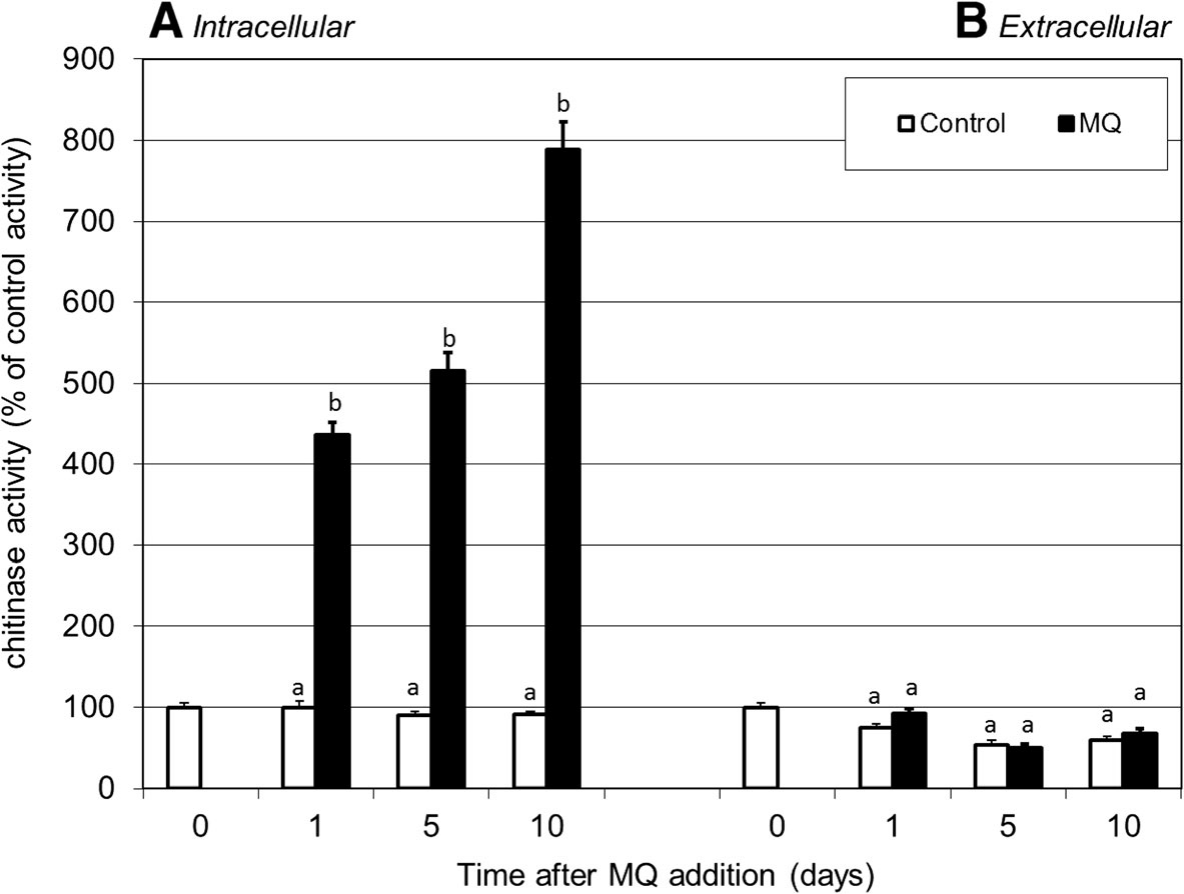
Effect of menadione (MQ) treatment on intra- (**a**) and extracellular chitinase activities (**b**) (expressed as % of the initial control values of samples collected just before prooxidants addition) in *P. pini* cultures. 14-day-old fungal cultures were treated with 0.75 mM of MQ solution. Cultures were collected at 1, 5, and 10 days after MQ addition. Data are mean ± SD for three measurements (*n* = 3). Values with *different letters* are significantly different (*p* ≤ 0.05)

Another element of the fungal hydrolytic system examined in this study was chitinase. Under the oxidative stress conditions described above, the activity of chitinase in the *P. pini* mycelium evidently increased compared to the control (Fig. 5). The highest enzyme activities were noted 10 days after the MQ addition (eight times higher than the control value). Chitin is known as a major component of fungal cell walls, which can be cleaved by the chitinase action. The results obtained suggest the possible participation of chitinase in the rebuilding of carbohydrate metabolism and probable changes in the structure of the fungal cell wall in response to oxidative stress conditions. It should be underlined that several applications of chitinases, e.g., production of single-cell proteins, isolation of fungal protoplasts, production of chitooligosaccharides, production of glucosamine and GlcNAc, estimation of fungal biomass, synthesis of artificial polysaccharides, or control of plant-pathogenic fungi have been reported [59]. Because the production and purification of chitinases, especially from microbial sources, have received considerable attention during recent decades, the present study fits well with these needs.

## Conclusions

According to recent literature, we examined for the first time the effect of menadione (MQ) addition on the natural biodegradation system in the medicinal white rot fungus *P. pini*. The menadione addition resulted in fluctuations of the antioxidative system, compared to controls (−MQ). The results obtained suggest that enhancement of the natural biodegradation metabolism is one of the strategies of adapting to oxidative stress conditions in this group of fungi. An important finding is that the increased activities of biotechnologically applicable enzymes (MnP, chitinase, and serine proteinases) indicate a novel method of stimulation of their activity. The issues emphasized in this work show that the presented way of oxidative stimulation could be a possible signal for rebuilding the fungal metabolism and that a very wide variety of cellular processes in fungi are regulated by prooxidative events.
